# Accouchement gémellaire en milieu africain: une analyse de 10 ans dans le district de Bamako au Mali: Twin deliveries in Africa: 10 years analysis in Bamako’s District in Mali

**DOI:** 10.11604/pamj.2018.29.21.14277

**Published:** 2018-01-10

**Authors:** Tiounkani Théra, Niani Mounkoro, Soumana Omar Traore, Albachar Hamidou, Mamadou Traore, Saleck Doumbia, Saoudatou Tall, Aminata Kouma

**Affiliations:** 1 Service de Gynécologie Obstétrique CHU du Point G, Bamako, Mali; 2 Service de Gynécologie Obstétrique CHU Gabriel Touré, Bamako, Mali; 3 Service de Gynécologie Obstétrique Centre de Référence Commune V de Bamako, Bamako, Mali; 4 Service de Gynécologie Obstétrique, CHU Kati, Bamako, Mali

**Keywords:** Accouchement gémellaire, pronostic, deuxième jumeau, Twin childbirth, second twin prognosis

## Abstract

**Introduction:**

Le but de cette étude était de déterminer les facteurs influençant le pronostic de l'accouchement du deuxième jumeau.

**Méthodes:**

L'étude était rétrospective et a porté sur tous les cas d'accouchements gémellaires enregistrés à la maternité du Centre de Santé de Référence de la commune V du District de Bamako du 1^er^Janvier 2007 au 31 Décembre 2016 soit sur une période de dix ans.

**Résultats:**

Nous avons enregistré 34.899 accouchements dont 1374 accouchements gémellaires soit une fréquence de 2,54%; près de 15% étaient des primipares; 39,16% étaient référées; 69,10% et 15,5% autres sans aucun suivi prénatal. Les facteurs influençant négativement pronostic du deuxième jumeau étaient: un délai ≥ 15minutes entre les jumeaux, le recours tardif à la césarienne, la non qualification de l'accoucheur, la grossesse monochoréale, le faible poids de naissance. Par contre la parité, la réalisation de manœuvres obstétricales n'avaient aucune influence sur le pronostic du deuxième jumeau.

**Conclusion:**

Le pronostic du deuxième jumeau est fortement influencé par un délai de naissance de plus de 15 minutes entre les jumeaux, les autres facteurs agissant comme des cofacteurs.

## Abstract (en)

**Introduction: Aim:**

It was to determine the factors influencing the prognosis of the second twin's delivery.

**Methods:**

The study was retrospective and covered all cases of twin births registered at our maternity from 1^st^January 2007 to 31^st^ December 2016 (10 years).

**Results:**

We recorded 34,899 deliveries among which 1374 twin deliveries (2.54%). Almost 15% were primiparous; 39.16% were referred; 69.10% and 15.5% without prenatal care. The factors negatively influencing prognosis for second twin were: a delay>15 minutes between twin deliveries, delayed caesarean section, non-qualification of the midwife, monochoreal, low birth weight. Otherwise parity and obstetric maneuvers had no influence on the prognosis 2^nd^ twin.

**Conclusion:**

The prognosis of 2^nd^ twinisstronglyinfluenced by a birth delay> 15 minutes, the other factors acting as cofactors.

## Introduction

la fréquence des grossesses gémellaires a considérablement augmenté ces dernières années depuis l'avènement des techniques d'Assistance Médicale sa la Procréation (AMP) dans les pays développés [1]. Cette augmentation est légèrement ressentie aujourd'hui en Afrique au sud du Sahara certainement à cause d'une amélioration de la couverture sanitaire dans certaines zones. La grossesse et l'accouchement gémellaire représentent une situation à haut risque et le deuxième jumeau reste exposé à des risques supplémentaires [1]. Pour certains auteurs ces risques sont surtout présents lorsque l'accouchement a lieu par voie basse et ce en fonction de plusieurs paramètres : la présentation, le mode d'accouchement, le terme, le poids, la différence de poids entre 1^er^ et le 2^nd^ jumeau. Nous avons mené cette étude dans le but d'évaluer le pronostic du 2^nd^ Jumeau selon un certains nombres de critères de jugement dont les principaux étaient la morbidité et la mortalité.

## Méthodes

Notre étude s'est déroulée au Centre de Santé de Référence de la Commune V (CS réf CV) du District de Bamako qui est une structure de niveau II de la pyramide sanitaire du Mali dont sa vocation première est la santé mère et de l'enfant. Nous avons réalisé une étude rétrospective s'étendant de la période allant du 1^er^Janvier 2007 au 31 Décembre 2016 soit une période de dix ans. L'étude a porté sur tous les cas d'accouchements gémellaires enregistrés dans le service. N'ont pas été incluses dans cette étude, les cas de grossesses gémellaires avec la mort de un ou des deux jumeaux. Ont servi de sources de collecte des données : le système d'information sanitaire locale (SISL), les registres d'accouchements, de comptes rendus opératoires, les registres de référenceset d'accouchement. Les données ont été saisies et analysées sur les logiciels Word et Excel 2000.

## Résultats

Durant la période d'étude, nous avons enregistré 34899 accouchements dont 1374 gémellaires (2,54%). Il s'agissait pour la plupart des patientes multipares avec un âge compris entre 20 et 34 ans. A noter que 15,50% des patientes n'avaient réalisé aucune consultation prénatale (Tableau 1). Les facteurs pronostics les plus péjoratifs étaient un délai > 15 mm entre la naissance du 1^er^ et du 2^ème^ jumeau, les petits poids de naissance (2500g). Par contre la parité, la réalisation de manœuvres obstétricales n'avaient aucune influence sur le pronostic du deuxième jumeau (Tableau 2). Par ailleurs l'absence d'accoucheur qualifié ainsi que la primiparité étaient des facteurs de mauvais pronostic. Par contre il n y avait pas de différence entre grossesses mono et bichoréales (Tableau 3).

**Tableau 1:** Profil sociodémographique des patientes

Variable étudié	Effectif	Pourcentage(%)
**Age (Années)**		
< 20	205	14,92
20-34	686	49,93
≥ 35	483	35,15
**Parité**		
Primipare	277	20,16
Paucipare	333	24,23
Multipare	764	57,61
**Suivi Prénatal**		
Oui	1161	84,50
Non	213	15,50
**Echographie**		
Oui	475	34,57

**Tableau 2:** Facteurs pronostiques du 2^ème^ jumeau

Variables étudiées	Mort-né		Morbide		Bon	
	Effectif	pourcentage	Effectif	pourcentage	Effectif	pourcentage
**Délai de naissance**						
≥ 15mn	28	2,04	43	2,13	178	12,9
< 15mn	8	5,58	13	0,25	1101	80,3
**Voie d’accouchement**						
Voie basse	11	0,80	54	3,93	1135	82,6
Voie Haute	6	0,44	13	0,95	155	11,2
**Manœuvre**						
Oui	20	1,45	40	2,9	753	54,8
Non	12	0,87	36	2,62	513	37,3
**Poids Nouveau-nés**						
< 2500g	33	2,40	57	4,15	592	43,1
≥ 2500g	7	0,51	14	1,02	671	48,8

**Tableau 3:** Facteurs pronostiques du 2ème jumeau

Variables étudiées	Mort-né		Morbide		Bon	
	Effectif	Pourcentage	Effectif	Pourcentage	Effectif	Pourcentage
**Choriocité**						
Monochorie	16	1,16	68	4,95	835	28,0
Bichoréal	16	1,16	51	3,70	838	60,9
Accoucheur						
**Qualifié**	13	0,95	38	4,95	1124	81,8
Non qualifié	20	1,46	36	2,62	143	10,4
**Parité**						
Primipare	13	0,95	22	1,60	297	21,6
Paucipare	9	0,65	24	0,74	395	28,7
Multipare	11	0,80	28	2,03	575	41,8

## Discussion

Les fréquences des accouchements gémellaires sont disparates, variant d'une région à l'autre. Des fréquences de 2,9% et 4,49% ont été rapportées respectivement par Moreira P et al. [2] à Dakar et Kouamé A et al. [3] au centre hospitalier universitaire (CHU) de Cocody à Abidjan. Par ailleurs ces fréquences étaient beaucoup plus faibles, en Tunisie avec 0,82% selon Dia M et al. [1]. Une patiente sur 4 (14,92%) avait moins de 20 ans, et une patiente sur 2 avait un âge compris entre 20 et 34 ans (49,93%). Les âges extrêmes étaient de 13 ans et 48 ans avec une moyenne d'âge de 28 +/- 4 ans. Dans une série ivoirienne les auteurs ont rapporté que 50,3% des patientes avaient un âge compris entre 26 et 35 ans avec un âge moyen de 27,8 ans et des extrêmes de 15 et 50 ans [3]. Dans notre série, les multipares ont représenté plus de la moitié de l'échantillon soit 55,61%, les paucipares 24,23% et les primipares 20,16%. Oger et al. [4] ont rapporté dans leur étude 29,1% de nullipares suivies des primipares et les paucipares avec chacune 27,98% des cas.

Nous avons noté que 84,50% (n =1161) des patientes avaient réalisé au moins une consultation prénatale et 77,30% de nos patientes étaient venues d'elles -mêmes. Une étude faite au CHU de Cocody en Cote d'Ivoire rapportait que 77,7% (n=150) des patientes étaient suivies dans les maternités périphériques et seulement 20,2% au CHU de Cocody et que 69,9% (n = 135) étaient évacuées ou référées [3]. Dans notre étude, seules 34,57% (n=475) avaient bénéficié d'une échographie et dans 40,18% (n = 552,) le diagnostic de la gémellité a été posé en salle d'accouchement par fois après la naissance du 1^er^ jumeau. Tous ces facteurs compliquent la prise en charge et aggravent davantage le pronostic du 2^ème^ jumeau surtout par le biais de l'allongement du délai de naissance entre le 1^er^ et le 2^nd^ jumeau ou par la pratique de manœuvres obstétricales sur le 2^nd^jumeau. Dans notre étude, 13 mort-nés et 22 cas de morbidités ont été enregistrées chez les primipares, 9 mort nés et 24 cas de morbidités chez les pauci pares et 11 mort nés et 28 cas de morbidités chez les multipares. Le mauvais pronostic pourrait être lié au délai de naissance prolongé entre les deux jumeaux étant donné que la plupart des femmes évacuées l'ont été après l'accouchement du premier jumeau. Kouamé A et al. [3] ont rapporté 18 cas de mort nés soit un taux de mortalité néonatale de 93%. Dans la même étude il a enregistré 43% de morbidités référées en néonatologie dont 51,8% de prématurés. Moreira P et al [2] à Dakar rapportait 7,86% de mauvais Apgar après 5 minutes chez les nouveaux nés de mères évacuées. Nous avons comparé l'état des nouveaux- nés pour des délais < 15 minutes et ≥ 15 minutes. Ainsi pour un délai < 15 minutes, nous avons enregistré 8 mort- nés contre 28 lorsque ce délai ≥ 15 minutes Aussi pour un délai < 15 minutes, nous avons enregistré, 8 mort- nés et 43 cas de morbidités pour un délai ≥ 15 minutes. Ces mêmes constats ont été rapportés dans la littérature portant sur le caractère morbide du deuxième jumeau né après un délai de 15 minutes [1,5]. Un délai moyen de 12,3 minutes avec des extrêmes de 1 minute et 412 minutes a été rapporté par Kouamé A et al. [3]. Ainsi, nous sommes d'avis que l'attitude active doit être la préféré pour terminer dans les plus brefs délais l'accouchement du deuxième jumeau [1,6]. Pour ce faire, l'accouchement gémellaire doit être effectué dans un milieu spécialisé par un accoucheur entrainé et qui maitrise les manœuvres obstétricales.

Notre étude laisse entrevoir que la voie d'accouchement du deuxième jumeau est aussi déterminante dans le pronostic de ce dernier surtout si cet accouchement tarde à se faire. Dans notre série 87,92%(n=1208) des 2^ème^ jumeaux sont nés par voie basse et 12,08% (n = 166) par voie haute. Ainsi nous avons enregistré 6 mort- nés et 13 cas de morbidités chez les enfants nés par césarienne contre 11 mort-nés et 54 cas de morbidités après accouchement par césarienne. La césarienne en elle-même n'est pas facteur de mauvais pronostic du 2^ème^ jumeau mais du recours tardif à cette dernière qui est en cause. Il pourrait s'agir de l'allongement de délai auquel fait suite la césarienne. Lembrouck C et al. [7] dans une étude avaient constaté que la baisse du taux de césarienne n'avait pas eu d'effet néfaste sur le devenir néonatal. Mottet N. et al. [8] dans leur série rapportent un taux global d'accouchement par voie basse de 54,7% et un taux de morbidité néonatale de 20,8% concernant essentiellement des enfants avec retard de croissance intra utérin. Konamé A. et al. [3] dans leur série rapportent que dans 51,3%des cas, l'accouchement du 2^nd^ jumeau s'est fait par voie basse. Oger AS et al. [4] ont rapporté un taux de césarienne de8,1% sur 2^nd^ jumeau. Ils notent une surmortalité néonatale relative des enfants grands prématurés inférieurs à 33 SA nés par voie basse (7 /40 ; 17,5% vs 5/62 ; 8,0%) mais que cette différence n'était pas significative. Schmitz T. et al. [5] dans leurs travaux n'ont pas mis en évidence une augmentation significative de la mortalité ou de la morbidité néonatale du 2^ème^ jumeau en cas de césarienne. Pour Schmitz T et al. [9], il apparait de plus en plus que de la morbidité et la mortalité du 2^ème^ jumeau soit fortement corrélée au risque de césarienne sur le 2^ème^ jumeau. Ces derniers avis sont largement partagés par Dhia et al. [1].

Les manœuvres obstétricales sont largement utilisées dans l'accouchement par voie basse du 2^ème^ jumeau. Elles sont, souvent, associées à l'allongement de délai de naissance du 2^ème^ jumeau. Ces manœuvre peuvent retentir sur le bien être fœtal et néonatal du 2^ème^ jumeau. Dans notre étude les manœuvres obstétricales ont été pratiquées dans 54,80% des cas (n = 753) avec 20 mort- nés et 40 cas de morbidités, contre 12 mort- nés et 36 cas de morbidités en l'absence de pratique de manœuvres obstétricales avec une différence statistiquement non significative. Dans la littérature internationale peu d'auteurs ont pratiqué des manœuvres obstétricales, car ont préconisé dans la quasi-totalité des cas la césarienne prophylactique si le 2^nd^ jumeau était en siège ou en transversale [4, 5,9]. Cependant, dans une étude portant sur l'accouchement avec des manœuvres sur le 2^nd^jumeau membranes rompues vs membranes intactes, Roesch M et al. [10] ont conclu que l'accouchement du 2^ème^ jumeau à membranes rompues ne grevait ni le pronostic maternel ni fœtal et n'augmentait pas le taux de césarienne sur le 2^ème^ jumeau. Kouamé A et al. [3] ont rapporté dans leur étude un cas de fracture du fémur suite à des manœuvres de grande extraction du siège sur le 2^nd^ jumeau.

Dans notre étude, un poids de naissance inférieur à 2500 grammes était un facteur de morbidité et létalité de élevée. En effet nous avons enregistré 33 mort-nés, 57 cas de morbidités pour un poids compris entre 2500 grammes contre 7 mort-nés et 14 cas de morbidités pour un poids supérieur à 2500 grammes avec. La prématurité a été un facteur aggravant de morbidité et de mortalité du 2^ème^ jumeau. Ainsi parmi les enfants de poids de naissance inférieur à 2500 grammes, 317 étaient les prématurés, 25 mort-nés et 41 cas de morbidités. La même tendance a été observée par certains auteurs [1, 3, 6,8]. Ainsi, Dhia Me et al [1] rapportaient que le faible poids de naissance < 1500 grammes était plus à risque de souffrance significative (p < 0,001). La détermination du type de chorionicité avant l'entrée en salle de travail est d'une importance capitale car peu guider l'attitude à observer vis-à-vis du 2^nd^ jumeau surtout en cas de monochorie, même pour ceux qui prônent le respect de la physiologie devant l'accouchement gémellaire. En effet en cas de monochorie, le décollement du placenta avant la naissance du 2^ème^ Jumeau est quasi constant, menaçant dangereusement par asphyxie le pronostic de celui-ci. Ainsi nous avons enregistré autant de mort-nés dans la monochorie que dans la bichorie soit 16 mort-nés dans chacun des cas. La morbidité était plus fréquemment observée dans la monochorie avec 68 cas contre 51 cas dans la bichorie. Aussi Roesch M et al. [6] rapportaient que les jumeaux monochoriaux présentaient des taux plus élevés d'hypotrophies en cas de grossesse menées à terme (p < 0,05) et de mortalité périnatale (p < 0,05) par rapport aux jumeaux bichoriaux. Dans leur travail, Oger AS et al. [4] ont confirmé qu'il y avait peu de différence de morbidité maternelle entre les grossesses gémellaires mono choriales et les grossesses gémellaires bi choriales; par contre la morbidité et la mortalité néonatale est constamment en faveur des grossesses gémellaires mono choriales. Tous les auteurs s'accordent à dire que l'accouchement gémellaire est un accouchement à haut risque encore plus pour le deuxième jumeau. Si l'accouchement du 1^er^ jumeau semble se dérouler comme celui de l'accouchement d'un fœtus unique; celui du 2^ème^ jumeau fait l'objet de beaucoup de débats et de controverses dont les attitudes varient d'une école à l'autre, d'un pays à l'autre et d'un continent à l'autre [1-10]. Nos résultats corroborent avec la tendance générale de la littérature. Ainsi nous avons enregistré 10 mort-nés et 36 cas de morbidités chez les nouveau-nés dont l'accouchement a été assisté par un personnel dit qualifié contre 20 mort-nés et 36 cas de morbidités pour les accouchements par personnel non qualifié.

## Conclusion

Nous sommes d'avis que l'accouchement du deuxième jumeau doit être actif afin de réduire le délai ou l'intervalle de naissance entre les deux jumeaux pour améliorer le pronostic de ce dernier. En dehors du délai de naissance entre les deux jumeaux, tous les autres facteurs nous semblent être des cofacteurs agissant par le biais de délai de naissance.

### Etat des connaissances actuelles sur le sujet

- Le pronostic de la grossesse gémellaire est plus mauvais que celui de la grossesse unique notamment à cause de la prématurité;
- Si le pronostic de la grossesse gémellaire est globalement mauvais, celui du 2^ème^ jumeaux est moins bon que celui du deuxième jumeau;
- Le pronostic de la grossesse gémellaire dépend en partie de l'efficacité de prise en charge des prématurés.

### Contribution de notre étude à la connaissance

- La parité et la réalisation de manœuvres obstétricales n'avaient aucune influence sur le pronostic du deuxième jumeau;
- L'absence d'accoucheur qualifié ainsi que la primiparité étaient des facteurs de mauvais pronostic;
- Il n y a pas de différence de pronostic entre grossesses mono et bichoréales.

## Conflits d’intérêts

Les auteurs ne déclarent aucun conflit d'intérêts.
